# Bilateral Plantar Verrucous Carcinoma

**Published:** 2016-12-07

**Authors:** Charlie Riccio, Kathryn King, Joshua B. Elston, Michael A. Harrington, C. Wayne Cruse

**Affiliations:** ^a^Department of Plastic Surgery, University of Tennessee Health Science Center, Memphis; ^b^Division of Plastic Surgery, Department of Surgery, University of South Florida Morsani College of Medicine, Tampa

**Keywords:** verrucous carcinoma, squamous cell carcinoma, plantar, human papilloma virus, malignancy

## DESCRIPTION

**Figure 1:**
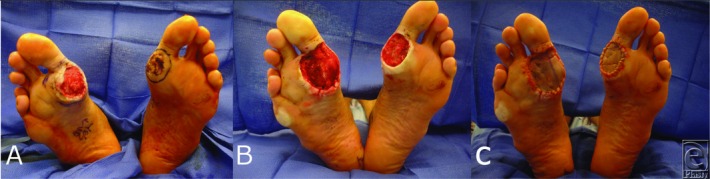
(a) Bilateral chronic plantar ulcerations diagnosed as verrucous carcinoma. (b) Appearance after tangential excision of the carcinomas. (c) Intraoperative view of thick-sheet split-thickness skin grafts to the bilateral plantar defects.

A 54 year-old male gentleman with a history of chronic ulcerations to the bilateral plantar feet presented with biopsy-proven verrucous carcinoma (VC). He underwent tangential excision, temporary closure with human skin allograft, and final reconstruction after clear permanent pathology margins with thick-sheet split-thickness skin grafting (Fig 1).

## QUESTIONS

1. **What is VC and how does it present?**
2. **How is VC diagnosed?**
3. **What are the predisposing factors associated with development of VC?**
4. **What is the preferred management of VC of the foot?**

## DISCUSSION

Verrucous carcinoma is a slow-growing, cauliflower-like, well-differentiated variant of squamous cell carcinoma (SCC) that typically affects men in the fourth to sixth decade of life. It was originally described in 1948 as slow-growing, wart-like lesions of the oral cavity.^1^ While these tumors rarely metastasized, they appeared to grow around lymphatic structures, rather than directly invading them.^1^ Since the original description, VC has been described as arising from the larynx, esophagus, anogenital region, palmar, and plantar surfaces.^2^ This report describes a patient with a history of chronic, bilateral plantar ulcerations and biopsies initially demonstrating keratoacanthoma-type SCC treated with laser therapy and topical 5-fluorouracil prior to referral to our clinic. Punch biopsies demonstrated hyperplastic squamous epithelium with focal human papillomavirus (HPV) infection. Subsequent excisional biopsies demonstrated bilateral verrucous SCC with positive peripheral and deep margins. Subsequent excision and grafting were performed after clear permanent pathological margins.

In 1976, 8 cases of wart-like growths on the plantar foot with well-differentiated keratinocytes were histologically described. It was proposed that these plantar lesions were histologically similar to the VC previously described by Ackerman.^3^ The majority of these lesions were treated as plantar warts for many years prior to biopsy, highlighting the delay between accurate diagnosis and treatment.^3^ Since plantar VC initially appears similar to the clinically benign plantar wart, biopsy of recalcitrant lesions is warranted to rule out more sinister processes. Punch, scallop, or excisional biopsies are necessary to evaluate the dermis for atypia as well as invasion.^2^ Microscopically, these lesions demonstrate both endophytic and exophytic growth of well-differentiated squamous epithelium with hyperkeratotic folds. Mild cytological atypia is common, and microabscesses may also be noted.^4^ Verrucous carcinoma can appear similar to verrucous skin lesions of diabetic neuropathy and is usually differentiated on the basis of invasion into local structures characteristic of the malignant potential of VC.^5^ Misdiagnosis is common in SCC with focal invasion or with giant condylomas, as they share similar architectural features.^6^

The pathogenesis of VC remains unclear. Reports have suggested association between tobacco use and oral VC and between previous trauma or chronic inflammation and cutaneous VC.^1^,^4^ There is controversy over the potential association between HPV infection and VC. A recent study found that only 7.7% of VC samples evaluated in the study were HPV positive, suggesting that while HPV may be associated with VC, the virus is unlikely the only causative agent.^6^

Currently, the most effective modality for treatment of VC is surgical excision.^3^,^5^ As it can be locally aggressive, it is important to obtain radiological studies prior to any procedure to ensure adequate excision. Radiotherapy is typically not advised, as it has historically been associated with transformation of oral VC into anaplastic lesions but may be utilized for those who cannot tolerate surgery or who refuse surgery.^7^ Imiquimod has been suggested as a possible treatment option, although more studies are warranted to validate its efficacy.^8^

**Figure 2:**
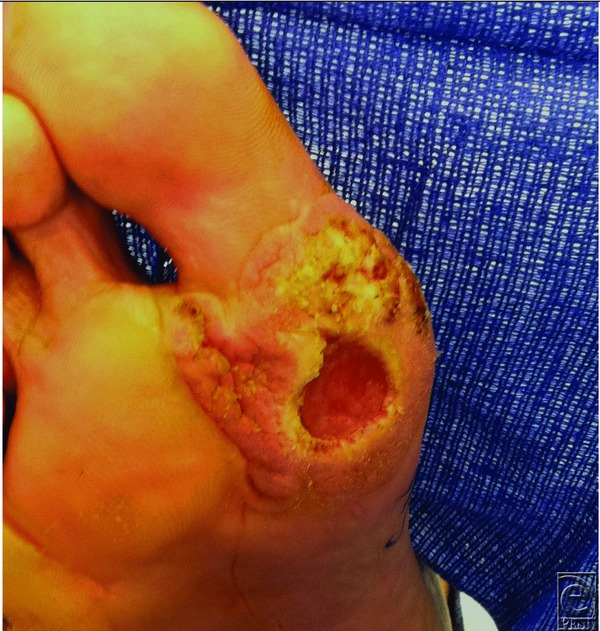
Close-up view of right plantar foot demonstrating verrucous (warty) appearance of initial lesion. Positive margins were encountered far outside of the area of the ulcer, supporting the aggressive nature of this carcinoma.

This case highlights the locally aggressive nature of VC despite initial clear margins on pathology (Fig 2). Accurate diagnosis of VC by a pathologist familiar with the disease and determining the extent of invasion are of paramount importance. Staging of VC follows the TNM American Joint Committee for Cancer staging guidelines for SCC.^2^ Verrucous carcinoma can be locally aggressive, and the most effective treatment modality to date is adequate surgical excision.^3^,^5^ Delay in treatment may lead to the need for more extensive surgical excision or amputation secondary to the invasive nature of these tumors. Radiation therapy should only be reserved for those unable to tolerate surgery or who refuse surgery.
